# Profound hyperacute cardiac allograft rejection rescue with biventricular mechanical circulatory support and plasmapheresis, intravenous immunoglobulin, and rituximab therapy

**DOI:** 10.1186/1749-8090-8-48

**Published:** 2013-03-16

**Authors:** David J Kaczorowski, Jashodeep Datta, Malek Kamoun, Daniel L Dries, Y Joseph Woo

**Affiliations:** 1 Division of Cardiovascular Surgery, University of Pennsylvania, 3400 Spruce Street, 6 Silverstein Pavilion, Philadelphia, PA, 19104, USA; 2 Department of Surgery, University of Pennsylvania, Philadelphia, PA, USA; 3 Department of Pathology and Laboratory Medicine, University of Pennsylvania, Philadelphia, PA, USA; 4 Department of Medicine, Cardiovascular Division, University of Pennsylvania, Philadelphia, PA, USA

**Keywords:** Heart failure, Heart failure operations, Heart transplant, Circulatory assist devices, Circulatory assistance (temporary)

## Abstract

Hyperacute rejection is a rare but potentially catastrophic complication after cardiac transplantation. We describe an unusual case of hyperacute rejection due to preformed anti-donor antibodies despite a negative preoperative panel-reactive antibody (PRA) screen. An excellent outcome was achieved in this case and our strategy involving the use of CentriMag ventricular assist devices (VADs) for biventricular support during treatment with rituximab, intravenous immunoglobulin (IVIG), and plasmapheresis is illustrated.

## Background

Hyperacute rejection (HAR) is a rare but dreaded complication following orthotopic heart transplantation with an approximate mortality rate of 70% [1,2]. It is mediated by preformed anti-donor antibodies and is characterized by complement deposition with widespread hemorrhage and thrombosis within the allograft [2]. In the modern era, HAR has largely been avoided by ensuring ABO compatibility between donor and recipient, and is restricted to cases in which preformed anti-HLA antibodies initiate antibody-mediated rejection [1]. To avoid this complication, panel-reactive antibody (PRA) screening is used to determine the presence of circulating antibodies to a random panel of donor lymphocytes. Along a continuum, a higher PRA is associated with worse rejection rates and poorer overall survival [3]. However, in spite of the recent advancements in PRA screening techniques, HAR can be occasionally encountered in the setting of a *negative* preoperative PRA. Fortunately, the incidence of such events is low, because the management of HAR after cardiac transplantation can be extremely challenging.

In instances of primary graft failure associated with HAR, temporary circulatory support with extracorporeal membrane oxygenation and intra-aortic balloon pump placement has been previously employed [2]. Further, immunologic strategies for eliminating circulating alloantibodies implicated in HAR include intravenous immunoglobulin (IVIG), monoclonal anti-CD20 antibody (rituximab), and plasmapheresis [2,4]. Here, we describe an unusual case of antibody-mediated HAR in a patient with a negative preoperative PRA. We also describe a successful strategy for allograft rescue utilizing biventricular CentriMag VAD support during targeted immunotherapy that resulted in an excellent outcome.

## Case presentation

A twenty-four year-old African American patient with non-ischemic cardiomyopathy underwent Heartmate II left ventricular assist device (LVAD) placement and tricuspid valve repair. No blood products were transfused at the time of LVAD placement. Approximately one year later, the patient developed a driveline infection and was listed for transplantation. Two months prior to transplantation, the patient required a blood transfusion. PRA screen was negative (0%) nine days after transfusion. Subsequent PRA seventeen days after transfusion was also negative (0%).

**Figure 1:**
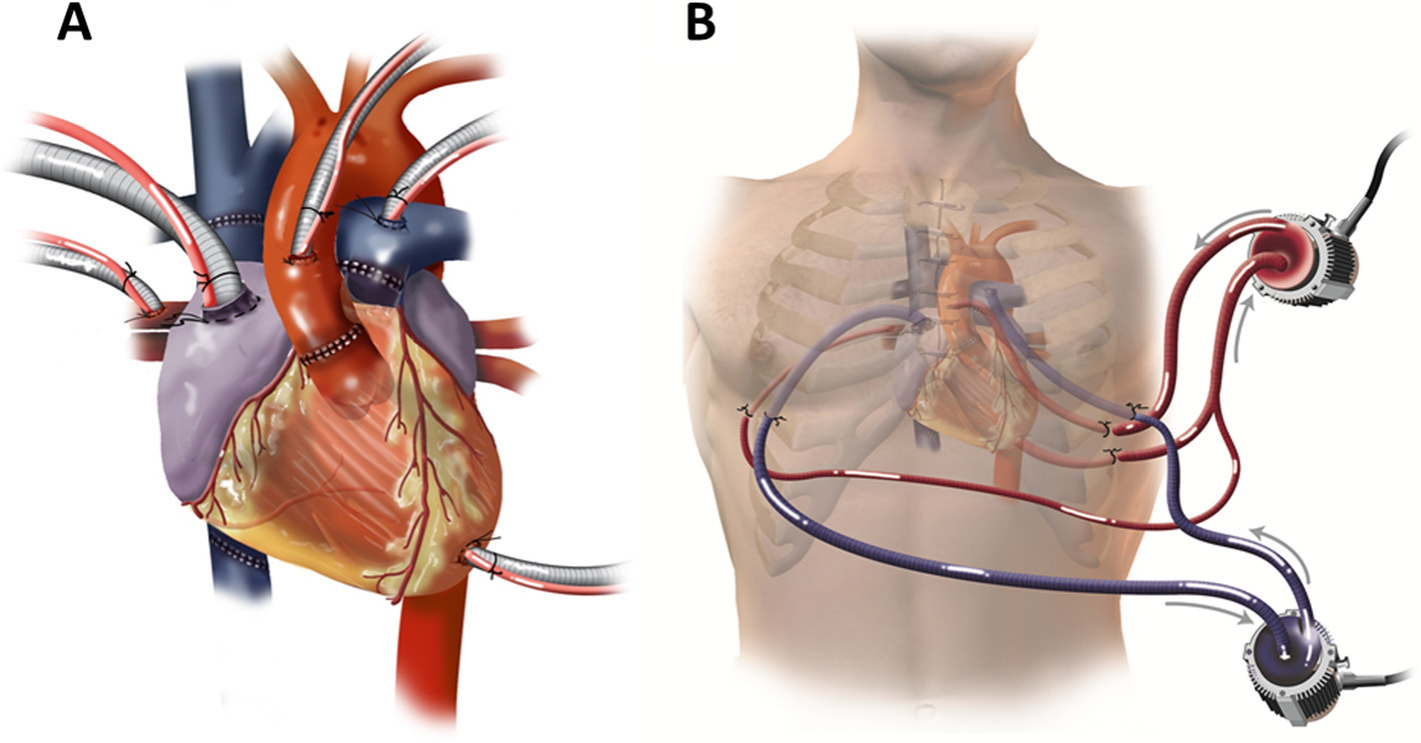
**(A) CentriMag VAD cannulation strategy.** A CentriMag VAD was used to support the left heart with cannulation via the left atrium, left ventricle, and aorta. Another CentriMag VAD was used to support the right ventricle with cannulation via the right atrium and pulmonary artery. This strategy allowed for excellent flows from both devices and complete decompression of the heart. **(B) CentriMag VAD access strategy.** All cannulas were brought out of the chest through intercostal or subcostal incisions, allowing closure of the sternotomy.

A donor heart of a compatible blood type (ABO class B) became available. The patient underwent redo-sternotomy, LVAD explantation, and bicaval orthotopic heart transplant using standard techniques with an ischemic time of 217 minutes. The heart failed to develop a spontaneous rhythm and severe biventricular failure developed. After two hours of reperfusion, cardiac function deteriorated further despite pharmacologic inotropic support and IABP placement. The heart was discolored and edematous with multiple petechiae. A CentriMag VAD was used to support the left heart with cannulation via the left atrium, left ventricle, and aorta. Another CentriMag VAD was used to support the right ventricle with cannulation via the right atrium and pulmonary artery (Figure 1A). All cannulas were brought out of the chest through intercostal or subcostal incisions, allowing closure of the sternotomy (Figure 1B).

**Figure 2:**
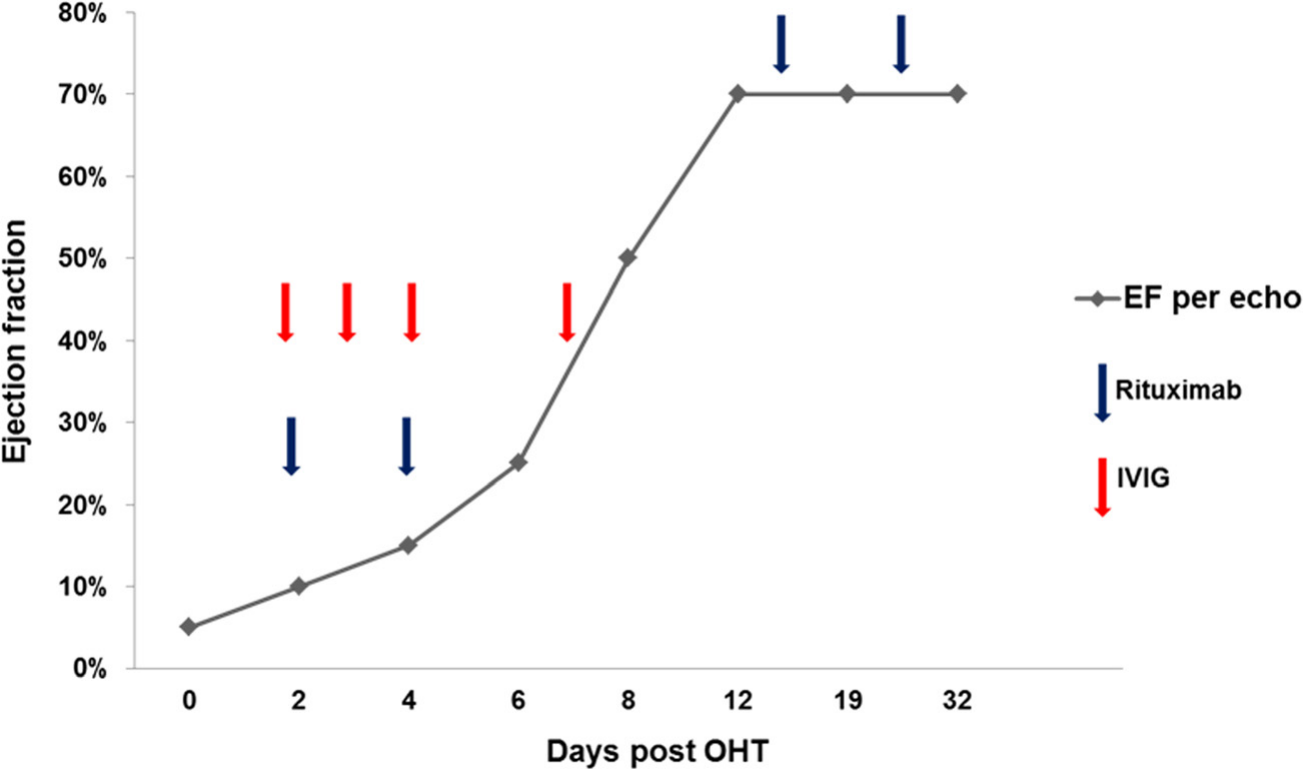
**Change in allograft function with post-operative immunomodulatory therapy.** Left ventricular ejection fraction is plotted as a function of time. The timing of administration of immunomodulatory agents is noted with arrows as indicated.

A postoperative retrospective crossmatch was positive. Treatment with rituximab, IVIG, and plasmapheresis was initiated in addition to a traditional regimen consisting of steroids, mycophenolate mofetil, and tacrolimus. Serial echocardiograms revealed improvement in ventricular function, ultimately demonstrating complete functional recovery (Figure 2). On post-operative day (POD) six, the patient was noted to have an acute increase in chest tube output and was taken back to the operating room for bleeding. However, at re-exploration, there was no evidence of ongoing bleeding. The graft demonstrated excellent biventricular function by both TEE and visual inspection. The patient was resuscitated overnight and the VADs were removed on POD seven. The immunotherapy regimen continued until POD twenty-four. Subsequent antibody screens demonstrated reduced reactivity to anti-donor antigen. Complete recovery of ventricular function was achieved and the patient was ultimately discharged to home forty-three days after transplantation.

## Conclusions

Risk factors for HAR in cardiac transplantation include high PRA, positive pre- or post-transplant crossmatch, induction therapy with OKT3, malignancy, and preceding infection [2]. Additionally, there is emerging evidence that VAD implantation can contribute to HLA sensitization by inducing secondary T-cell activation and B-cell reactivity, thereby increasing the risk for antibody-mediated rejection [3]. However, even VAD-bridged patients who are preoperatively identified as being highly sensitized may be candidates for cardiac transplantation. Lick *et al.* recently demonstrated that cardiac transplantation of LVAD patients with high PRA without preoperative crossmatch using on-bypass plasmapheresis and alemtuzumab resulted in equivalent midterm survival compared with non-sensitized patients [5].

PRA screening practices, crossmatch determinations, and management of sensitized patients vary considerably among institutions. Moreover, all PRA screening modalities are not created equal. Traditional techniques that are more commonly used at most institutions include cell-based complement-dependent cytotoxicity (CDC) and anti-human globulin-augmented lymphocytotoxicity assays [6]. However, newer modalities such as enzyme-linked immunosorbent (ELISA) and flow cytometric (FCM) assays appear to be more sensitive in their ability to detect class II antigens than the older techniques. Furthermore, FCM-detectable pre-transplant antibodies better differentiated sensitized versus non-sensitized patients, and predicted allograft rejection more reliably than the CDC method [7]. Our institution employs Luminex single antigen bead assays for HLA antibody screening.

Once antibody-mediated rejection has been identified, options for treatment include plasmapheresis, immunoadsorption, IVIG, cyclophosphamide administration, increasing doses of immunosuppression, and rituximab [2,4]. Rituximab is a chimeric humanized monoclonal antibody against the pan-B cell surface molecule CD20 that has showed promise in treating antibody-mediated rejection in cardiac transplantation as well as inadvertent ABO-incompatible lung transplantation [4]. Although rituximab induces a rapid depletion of CD20-expressing B cells in peripheral blood, it has little or no effect on circulating antibodies. Therefore, several reports have advocated for complementing the use of rituximab with modalities that deplete circulating antibodies such as plasmapheresis, IVIG, or immunoadsorption. Other treatment options reported in the literature include cyclophosphamide, OKT3, and anti-thymocyte globulin [4]. In our patient, the use of IVIG and plasmapheresis as an adjunct to rituximab resulted in an excellent outcome.

In addition to the use of appropriate immunotherapy targeting antibody-mediated rejection, the employment of biventricular CentriMag VAD support was critical in achieving complete functional recovery in this case. The cannulation strategy employed here allowed optimal decompression of both left and right sides of the heart during recovery. In particular, cannulating the left side of the heart through both the LV apex and the right superior pulmonary vein ensured complete decompression of the left ventricle. Excellent flows were achieved allowing ongoing support of organ function. Since heparin was not used in the early post-operative period, early bleeding complications were avoided. Since the sternum was able to be closed, infection and sternal wound complications were also avoided in this heavily immunosuppressed patient. Avoidance of early bleeding, preventing complications of an open sternum, and successful ongoing support of allograft function while the heart was being rescued allowed for a successful outcome.

## Consent

Written informed consent was obtained from the patient for publication of this Case report and any accompanying images. A copy of the written consent is available for review by the Editor-in-Chief of this journal.

## Abbreviations

CDC: Cell-based complement-dependent cytotoxicity; ELISA: Enzyme-linked immunosorbent and FCM flow cytometric; HAR: Hyperacute rejection; IVIG: Intravenous immunoglobulin; PRA: Panel-reactive antibody; POD: Post-operative day; VADs: Ventricular assist devices

## Competing interests

The authors have no competing interests to declare.

## Authors’ contributions

DJK: intra-operative and post-operative care of the patient, manuscript drafting and editing. JD: post-operative care of the patient, manuscript drafting. MK: post-operative immunologic evaluation and monitoring, manuscript drafting. DLD: pre-/post-operative care of the patient, manuscript drafting. YJW: intra-operative and pre-/post-operative care of the patient, manuscript drafting and editing. All authors read and approved the final manuscript.
